# Single Shot Lensless Interferenceless Phase Imaging of Biochemical Samples Using Synchrotron near Infrared Beam

**DOI:** 10.3390/bios12121073

**Published:** 2022-11-24

**Authors:** Molong Han, Daniel Smith, Soon Hock Ng, Tomas Katkus, Aravind Simon John Francis Rajeswary, Periyasamy Angamuthu Praveen, Keith R. Bambery, Mark J. Tobin, Jitraporn Vongsvivut, Saulius Juodkazis, Vijayakumar Anand

**Affiliations:** 1 Optical Sciences Centre and ARC Training Centre in Surface Engineering for Advanced Materials (SEAM), School of Science, Swinburne University of Technology, Hawthorn, VIC 3122, Australia; 2 Institute of Physics, University of Tartu, W. Ostwaldi 1, 50411 Tartu, Estonia; 3 Infrared Microspectroscopy (IRM) Beamline, ANSTO—Australian Synchrotron, Clayton, VIC 3168, Australia; 4 Tokyo Tech World Research Hub Initiative, School of Materials and Chemical Technology, Tokyo Institute of Technology, 2-12-1, Ookayama, Meguro-ku, Tokyo 152-8550, Japan

**Keywords:** phase imaging, bioimaging, synchrotron, near infrared beam, holography, incoherent optics, chemical imaging, phase retrieval, 3D imaging

## Abstract

Phase imaging of biochemical samples has been demonstrated for the first time at the Infrared Microspectroscopy (IRM) beamline of the Australian Synchrotron using the usually discarded near-IR (NIR) region of the synchrotron-IR beam. The synchrotron-IR beam at the Australian Synchrotron IRM beamline has a unique fork shaped intensity distribution as a result of the gold coated extraction mirror shape, which includes a central slit for rejection of the intense X-ray beam. The resulting beam configuration makes any imaging task challenging. For intensity imaging, the fork shaped beam is usually tightly focused to a point on the sample plane followed by a pixel-by-pixel scanning approach to record the image. In this study, a pinhole was aligned with one of the lobes of the fork shaped beam and the Airy diffraction pattern was used to illuminate biochemical samples. The diffracted light from the samples was captured using a NIR sensitive lensless camera. A rapid phase-retrieval algorithm was applied to the recorded intensity distributions to reconstruct the phase information. The preliminary results are promising to develop multimodal imaging capabilities at the IRM beamline of the Australian Synchrotron.

## 1. Introduction

Fourier transform infrared (FTIR) spectroscopy has become one of the widely used molecular fingerprinting method over the years providing structural, functional and compositional information of biochemical samples [1]. Most of the commercially available FTIR systems can measure the absorption spectrum of the samples, but cannot obtain the spatio-spectral image. One of the main reasons for the lack of imaging capabilities is the lack of sensor technology that can detect intensity variations over the entire range 3–20 μm. Besides, most of the FTIR systems use an internal thermal (Globar™) IR source. The FTIR microspectroscopy system coupled to a synchrotron light source offer the solution to the above problem. In this study, we mainly focused on the optics and beamline configuration at the Australian Synchrotron IRM beamline [2]. The synchrotron-IR beam has a high brightness. The IRM system at the Australian Synchrotron is equipped with a liquid nitrogen cooled single-pixel mercury-cadmium-telluride (MCT) detector and a liquid nitrogen cooled focal plane array (FPA) imaging detector with 64 × 64 pixels, both possessing a broad sensitivity in mid-IR spectral region suitable for many applications. The IRM system uses the single-pixel MCT detector with a pixel-by-pixel scanning approach to record intensity images. For small objects, i.e., with a low field-of-view (FoV), the FPA imaging detector can be used with a single camera shot.

In recent years, novel imaging experiments have been attempted beyond the physical boundaries using the IRM system at the Australian Synchrotron [3,4,5]. A single shot 3D semi-synthetic imaging technique has been demonstrated recently at the IRM beamline using the FPA-FTIR imaging system, with synchrotron source, and a computational algorithm to exploit the spatio-spectral aberrations [3]. However, the experimental demonstration of a single shot 3D imaging using synchrotron beam is challenging due to the unique nature of the synchrotron-IR beam consisting of a fork shaped intensity distribution. Most of the FTIR experiments performed at the IRM beamline are carried out using the single-pixel MCT detector and a pixel-by-pixel scanning of a tightly focused beam with a 36× Schwarzschild IR reflecting objective lens (NA = 0.5). Therefore, the developed computational method was experimentally demonstrated in the replica microspectroscopy system with an internal Globar™ IR source and the FPA imaging detector on the offline instrument at the Australian Synchrotron IRM beamline [4]. Another significant research outcome was the diffraction-limited anisotropy mapping using the synchrotron-IR source on the online instrument of the IRM beamline in the scanning approach [5].

The development of multimodal imaging has proven useful for various applications such as cancer diagnosis and treatment [6,7]. Digital holography methods have been developed to achieve multimodal imaging consisting of quantitative phase imaging and fluorescence imaging [8,9,10]. The previously mentioned methods will be highly valuable if implemented at the IRM beamline as they can reveal additional information for connecting responses from different properties of the sample such as structural, functional, phase and compositional information. Nevertheless, the above approaches involve complicated optical configurations [8,9,10], making them unsuitable for an already complicated synchrotron-IR microspectroscopy system. Besides, a coherent illumination source is needed in order to record the phase information. Recently, a lensless, interferenceless 3D quantitative phase imaging technique has been developed based on a phase-retrieval algorithm with a rapid convergence of less than five iterations with an incoherent source with a low temporal coherence [11]. This configuration is extremely simple for any 3D phase imaging application with a minimum requirement of a uniform optical beam to illuminate the samples. It is however necessary to consider the fact that the synchrotron-IR beam has a unique fork shaped intensity distribution, which is challenging to implement even for intensity imaging without tight focusing and pixel-by-pixel scanning. 

In this study, we present a novel approach for phase imaging using the synchrotron-IR beam. Our approach results from two aspects of the IRM system of the Australian Synchrotron. Firstly, even though the IR beam extracted from the synchrotron has a broad spectral range, not all the wavelengths are applied for spectroscopy applications. It was observed that the FTIR microspectroscopy system exhibits an improvement of signal-to-noise ratio (SNR) in the high-wavenumber spectral region by a factor of four when the beam at the wavenumbers higher than 3950 cm^−1^, which is predominantly in the near-IR (NIR) region, was filtered out. Therefore, the NIR spectral region of the synchrotron-IR beam is usually blocked using a 3950 cm^−1^ low-pass filter during measurement of the samples. Besides, the above method does not affect the performance of the system. In this research, by removing this filter, we have successfully utilized the synchrotron NIR beam for phase imaging. Secondly, the nature of the beam, whose intensity distribution has been extensively discussed earlier. In addition to the intensity distribution, the beam exhibited a characteristic polarization property consisting of linear and circular polarizations and also spatial and temporal incoherence [12]. Therefore, implementing an interference-based approach with the above beam will result in unpredicted responses. Consequently, the lensless, interferenceless phase imaging approach is found to be ideal for this synchrotron beam configuration. The quantitative phase distribution along with the spectral images may help understand the samples at a deeper level.

## 2. Materials and Methods

**Figure 1:**
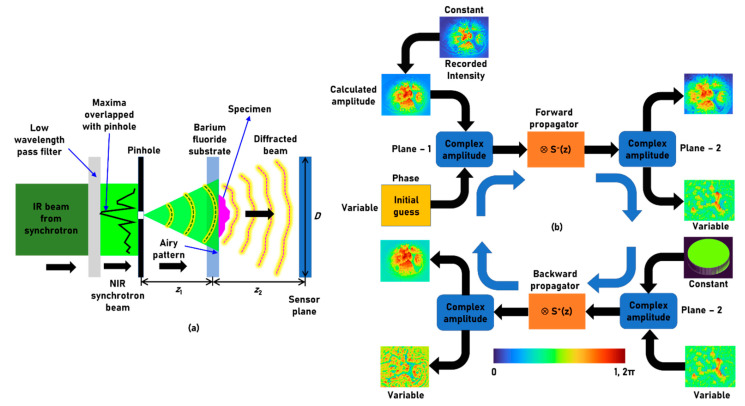
(**a**) Schematic of the optical configuration. (**b**) Phase-retrieval algorithm with spherical propagator for estimation of phase at plane 2 from the intensity at plane 1. ‘⊗’ – 2D convolutional operator.

The optical configuration of the NIR phase imaging module of the Australian Synchrotron IRM beamline is shown in Figure 1a. The synchrotron-IR beam extracted from the storage ring is filtered to allow only the low wavelength region with a cut-off at the NIR region. A pinhole was aligned with the collimated beam with one of the two lobes at a point corresponding to the intensity maxima. The light diffracted from the pinhole creates an Airy intensity pattern, which is used for illuminating the object. The light from the pinhole can be approximated to a point object with a Kronecker Delta like function with an amplitude $\sqrt{I_{o}}$. The complex amplitude of the Airy pattern obtained at a distance of *z*_1_ from the pinhole can be given as $C_{1}\sqrt{I_{o}}Q\left(1/z_{1}\right)$, where *C*_1_ is a complex constant and $\;Q\left(a\right)=\exp\left[j\pi aR^{2}/\lambda \right]$ where $R={\left(x^{2}+y^{2}\right)}^{1/2}$. A specimen is mounted on a barium fluoride (BaF_2_) substrate with a thickness profile of *t*(*x*,*y*). The phase difference generated by the specimen is given as $\Phi_{s}\left(x,y\right)=\frac{2\pi t\left(x,y\right)}{\lambda }\left[n\left(x,y\right)-1\right]$, where *n*(*x*,*y*) is the refractive index variation of the sample in space. The complex amplitude immediately after the specimen can be expressed as $C_{2}\sqrt{I_{o}}Q\left(1/z_{1}\right)\exp\left[-j\Phi_{s}\left(x,y\right)\right]$, where *C*_2_ is a complex constant. The light modulated by the specimen is propagated by a distance of *z*_2_ to reach the image sensor. The intensity distribution recorded by the sensor is given as $I_{s}={\left|C_{2}\sqrt{I_{o}}Q\left(1/z_{1}\right)\exp\left[-j\Phi_{s}\left(x,y\right)\right]⊗Q\left(1/z_{2}\right)\right|}^{2}$, where ‘⊗’ is a 2D convolutional operator.

The next step is to extract phase information in the specimen plane from the intensity information available at the sensor plane. Assuming that the intensity available at the specimen plane is a constant, there are two unknown parameters namely the phases at the sensor plane and specimen plane. The phase information at the sensor plane is usually obtained by interfering the object wave from the sample with a reference wave. In the case when the phase information is not available, it can be estimated using Gerchberg-Saxton algorithm (GSA) [13,14,15,16]. There are two complex amplitudes ψ_1_ and ψ_2_ corresponding to the two planes: specimen plane and sensor plane with two unknowns, which are the phases at those planes. The GSA is shown in Figure 1b. The complex amplitude ψ_2_ given as $\sqrt{I_{s}}$ (phase = 0) is propagated from sensor plane to the specimen plane using spherical propagator given as $S^{-}\left(z\right)=\exp\left[-j2\pi R/\lambda \right]$, where $R=\sqrt{x^{2}+y^{2}+z^{2}}$ generating a complex amplitude given as $S^{-}\left(z\right)⊗\sqrt{I_{s}}$ at the specimen plane. At the specimen plane, the amplitude is known but the phase is unknown. So, the complex amplitude is modified as $\sqrt{I_{O}}.exp\left[j\left(arg\left\{S^{-}\left(z\right)⊗\sqrt{I_{s}}\right\}\right)\right]$ in the specimen plane, where $I_{O}$ is a constant function. The complex amplitude $\psi_{1}=\sqrt{I_{O}}.exp\left[j\left(arg\left\{S^{-}\left(z\right)⊗\sqrt{I_{s}}\right\}\right)\right]$ is propagated to the sensor plane using the spherical propagator given as $S^{+}\left(z\right)=\exp\left[j2\pi R/\lambda \right]$. The complex amplitude at the sensor plane is given as $\psi_{2}=\left\{\sqrt{I_{O}}.exp\left[j\left(arg\left\{S^{-}\left(z\right)⊗\sqrt{I_{s}}\right\}\right)\right]\right\}⊗S^{+}\left(z\right)$ whose amplitude is replaced by $\sqrt{I_{s}}$ and phase is retained and carried-on to the next iteration. This process is continued until the phase of the specimen is estimated. The numerical aperture (NA) of the imaging system is given as ~*D*/2*z*_2_, where *D* is the diameter of the image sensor given as *N* × Δ, where *N* is the number of pixels in the sensor and Δ is the pixel size. The magnification is given as *M* = 1 + *z*_2_/*z*_1_. The lateral and axial resolutions of the imaging system are given as ~λ/NA and ~λ/NA^2^, respectively. By changing the value of *z* in the GSA, it is possible to estimate phase distributions corresponding to different planes. 

## 3. Experiments

**Figure 2:**
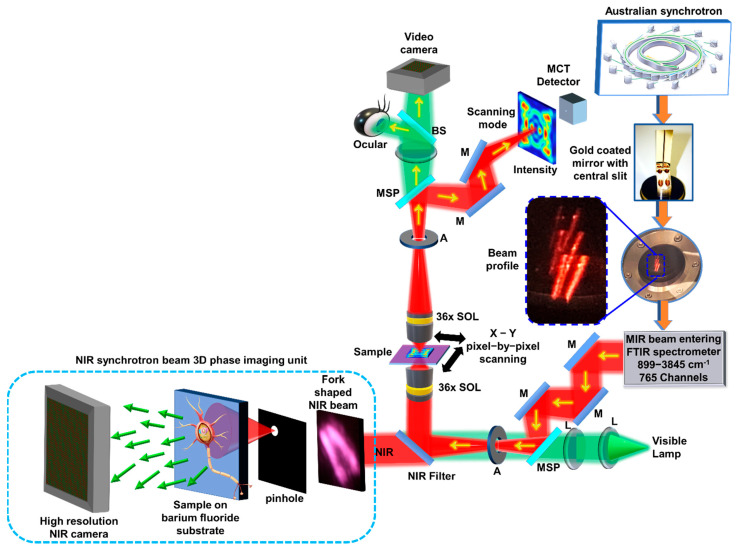
Optical configuration of the IRM system at the Australian Synchrotron and the newly attached synchrotron NIR phase imaging module shown within dotted line. BS—beam splitter, M—mirror, L—lens, MSP—motorized sliding plate, A—aperture, MIR—mid infrared. The synchrotron-IR beam is extracted using the gold coated mirror with central slit, and enters the FTIR spectrometer, which is subsequently coupled into the IR/VISIBLE transmission microscope.

The synchrotron-IR beam extracted from the storage ring using a gold-coated mirror with a central slit has a fork shaped intensity distribution [17]. Light from a visible lamp is aligned collinearly with the synchrotron-IR beam for alignment of the sample during beam alignment in the microscope. For most experimental conditions, the fork shaped beam is tightly focused to a point on the sample plane using a 36× Schwarzschild IR reflecting condenser (NA = 0.5). After passing through the sample, the beam is collected by an identical 36× (NA = 0.5) Schwarzschild IR reflecting objective and is focused on to a single pixel MCT detector as shown in Figure 2. The spectral data collection is moved from one point to the other until completing a map, and the information thus collected by the MCT detector is combined to obtain the full chemical image using the OPUS software (Bruker Optik GmbH, Ettlingen, Germany). For the experiment reported here, a high frequency pass filter is inserted in the path of the beam to allow the low wavelength region with a cut-off at the NIR region of the synchrotron beam. The NIR phase imaging module, shown in the dotted box of Figure 2, consists of a pinhole with a diameter of 200 μm, a BaF_2_ substrate with a thickness of ~1 mm and 2.5 cm diameter containing the specimen and a NIR sensitive camera-Canon EOS 6D (5568 × 3708) pixels without lenses and with a pixel pitch of 6.5 μm. The module is carefully built close to the IR microscope while the camera was mounted onto a tripod. The pinhole was aligned with the NIR beam to match the intensity maxima with the pinhole to achieve a maximum throughput. The diffracted beam was allowed to expand such that the diameter of the central maxima of the Airy pattern was ~6 mm. The substrate with specimen was mounted at this plane and aligned with the central maxima of the Airy pattern. The image sensor was mounted at a distance of ~7 cm from the sample. The NA of the system along *x* and *y* directions are 0.25 and 0.17, respectively. The lateral resolutions of the system along the *x* and *y* directions are 3.8 μm and 5.8 μm, respectively. However, the pixel size 6.5 μm samples the information and therefore the secondary resolution limit is 6.5 μm. The axial resolution along the *x* and *y* directions are approximately 15 μm and 34 μm, respectively. 

**Figure 3:**
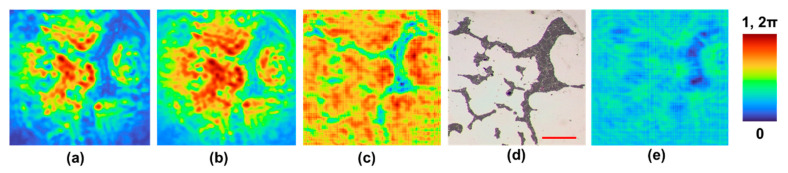
(**a**) Recorded intensity distribution for the latex beads (~15 μm in diameter). (**b**) Calculated amplitude of the recorded intensity distribution. (**c**) Reconstructed phase image obtained from the phase-retrieval algorithm after two iterations. (**d**) Reference image recorded using an optical microscope. (**e**) Estimated phase image at the sensor plane. The scale bar is approximately 1 mm.

Two samples were prepared for the study. The first sample consists of randomly arranged latex beads each with an average diameter of ~15 μm. The second sample consists of a section of a wing of an insect. The diffracted intensity distribution from the polymer bead sample was recorded using the camera. The phase retrieval algorithm was run with a distance of 7 cm and number of iterations of four. Figure 3a–e demonstrate the image of the recorded intensity distribution for the polymer bead sample, calculated amplitude of the recorded pattern, reconstructed image using phase-retrieval algorithm after two iterations, reference image recorded using an optical microscope and the phase estimated at the sensor plane, respectively. The typical features of the sample as seen in Figure 3d is also visible in Figure 3c. The resolution of the reconstructed image is lower than that of the reference image obtained from the optical microscope as the wavelength is lower in the case of visible microscope. Since the sample is a thin sample with all beads in the same plane, the variation of the depth in the phase-retrieval algorithm did not create any change between the elements of the object.

**Figure 4:**
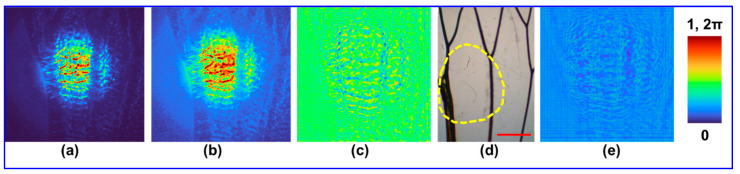
(**a**) Recorded intensity distribution for the wing sample taken from an insect. (**b**) Calculated amplitude of the recorded intensity distribution. (**c**) Reconstructed phase image obtained from the phase-retrieval algorithm after four iterations. (**d**) Reference image recorded using an optical microscope. (**e**) Estimated phase image at the sensor plane. The yellow dotted lines in (**d**) indicates the region of beam illumination. The scale bar is approximately 1 mm.

The second sample was an insect wing. The captured diffraction pattern is shown in Figure 4a and the calculated amplitude is shown in Figure 4b. The reconstructed image obtained from the phase-retrieval algorithm after four iterations is shown in Figure 4c. The image of the wing captured by an optical microscope and the area of study is shown in Figure 4d. The phase calculated at the sensor plane is shown in Figure 4e. The veins of the wings absorb NIR, which is seen as blue colour in the reconstructed image. Phase variations are seen within the transparent regions of the wings, which are not visible in the optical microscope.

## 4. Discussion

The reconstruction results obtained show the possibility of phase imaging using the synchrotron NIR beam, which is usually being filtered out. One of the challenges associated with this experiment was that most of the samples used exhibited a strong absorption in the NIR region and therefore weak signals were observed in the region indicated by a valley. The reconstruction result of the polymer bead sample was found to be consistent with its corresponding optical microscopic image, except for the resolution due to the larger wavelengths, lower spatial coherence and lower numerical aperture. The reconstruction result of the insect wing revealed additional information than what was obtained from the optical microscope. In addition to the branches of the wings, the phase variation within the transparent regions of the wing indicated the thickness profile. 

## 5. Summary and Conclusions

The synchrotron-IR beam at the Australian Synchrotron IRM beamline has a unique fork shaped intensity distribution, which when refocused using high NA reflecting optics allows for pixel-by-pixel scanning based imaging method with a single pixel MCT detector. The NIR part of the synchrotron-IR beam is usually filtered out to improve the SNR of spectral imaging. In this study, the opportunities mentioned were exploited to apply a rapid phase-retrieval algorithm for phase imaging using the NIR spectral range of the synchrotron-IR beam. To the best of our knowledge, this is the first phase imaging result obtained from the Australian Synchrotron from a single camera shot [18,19,20]. Two different samples, including polymer beads dried on a BaF_2_ substrate and an insect wing were used to demonstrate the capability of the new NIR approach described here. In the case of polymer beads, there was a significant overlap between the reconstructed image and reference image obtained from the optical microscope. In the case of the second sample, additional information related to the phase variation in the transparent region of the insect wings was visible. A larger pinhole ~200 μm was used to trade-off spatial coherence for obtaining higher light throughput. The quality of the results is affected by the low spatial coherence. Some recent studies indicated the possibilities of employing a single refractive lens without two beam interference for 3D imaging with a single camera shot [21]. We believe that the proposed method is even better as it is lensless, interferenceless and has potential to image 3D phase information.

We believe that the developed technique if integrated to the current measurement system of the IRM beamline, operating in the mid-IR, will benefit understanding of the specimens better as the functional information can be correlated with the phase information. It must be noted that even if the fork shaped beam is converted into a uniform illumination, achieving single shot imaging capability is only possible with an FPA imaging detector. However, with the mid-IR FPA imaging detector available at the Australian Synchrotron beamline, the resolution is highly limited as it consists of only 64 × 64 pixels. In the near future, we plan to explore the possibility of overlaying in real time the measured intensity distributions obtained with the scanning approach and the phase distributions obtained from the proposed method, to generate multimodal images.

## Data Availability

The data and MATLAB codes can be obtained from the authors upon reasonable request.

## References

[1] Haas J., Mizaikoff B. (2016). Advances in mid-infrared spectroscopy for chemical analysis. Annu. Rev. Anal. Chem..

[2] Tobin M., Vongsvivut J., Martin D., Sizeland K., Hackett M., Takechi R., Fimorgnari N., Lam V., Mamo J., Carter E. (2018). Focal plane array IR imaging at the Australian Synchrotron. Infrared Phys. Technol..

[3] Anand V., Ng S.H., Katkus T., Maksimovic J., Klein A., Vongsvivut J., Bambery K., Tobin M.J., Juodkazis S. (2021). Exploiting spatio-spectral aberrations for rapid synchrotron infrared imaging. J. Synchrotron. Rad..

[4] Anand V., Han M., Maksimovic J., Ng S.H., Katkus T., Klein A., Bambery K., Tobin M.J., Vongsvivut J., Juodkazis S. (2022). Single-shot mid-infrared incoherent holography using Lucy-Richardson-Rosen algorithm. Opto-Electron. Sci..

[5] Ryu M., Honda R., Balcytis A., Vongsvivut J., Tobin M.J., Juodkazis S., Morikawa J. (2019). Hyperspectral mapping of anisotropy. Nanoscale Horiz..

[6] Tempany C.M., Jayender J., Kapur T., Bueno R., Golby A., Agar N., Jolesz F.A. (2015). Multimodal imaging for improved diagnosis and treatment of cancers. Cancer.

[7] Kim J., Piao Y., Hyeon T. (2009). Multifunctional nanostructured materials for multimodal imaging, and simultaneous imaging and therapy. Chem. Soc. Rev..

[8] Kumar M., Quan X., Awatsuji Y., Tamada Y., Matoba O. (2020). Digital Holographic Multimodal Cross-Sectional Fluorescence and Quantitative Phase Imaging System. Sci. Rep..

[9] Hai N., Rosen J. (2020). Doubling the acquisition rate by spatial multiplexing of holograms in coherent sparse coded aperture correlation holography. Opt. Lett..

[10] Hai N., Rosen J. (2020). Phase contrast-based phase retrieval: A bridge between qualitative phase contrast and quantitative phase imaging by phase retrieval algorithms. Opt. Lett..

[11] Anand V., Katkus T., Linklater D.P., Ivanova E.P., Juodkazis S. (2020). Lensless Three-Dimensional Quantitative Phase Imaging Using Phase Retrieval Algorithm. J. Imaging.

[12] Ryu M., Linklater D., Hart W., Balčytis A., Skliutas E., Malinauskas M., Appadoo D., Tan Y.R.E., Ivanova E.P., Morikawa J. (2018). 3D printed polarizing grids for IR-THz synchrotron radiation. J. Opt..

[13] Gerchberg R.W., Saxton W.O. (1972). A practical algorithm for the determination of phase from image and diffraction plane pictures. Optik.

[14] Fienup J.R. (1982). Phase retrieval algorithms: A comparison. Appl. Opt..

[15] Bauschke H.H., Combettes P.L., Luke D.R. (2002). Phase retrieval, error reduction algorithm, and Fienup variants: A view from convex optimization. J. Opt. Soc. Am. A.

[16] Bao P., Zhang F., Pedrini G., Osten W. (2008). Phase retrieval using multiple illumination wavelengths. Opt. Lett..

[17] Cheeseman S., Truong V., Vongsvivut J., Tobin M.J., Crawford R., Ivanova E.P. (2019). Applications of Synchrotron-Source IR Spectroscopy for the Investigation of Insect Wings.

[18] Ozcan A., McLeod E. (2016). Lensless imaging and sensing. Annu. Rev. Biomed. Eng..

[19] Göröcs Z., Ozcan A. (2012). On-chip biomedical imaging. IEEE Rev. Biomed. Eng..

[20] Kumar R., Hai N., Rosen J. (2022). Single-shot TIE using polarization multiplexing (STIEP) for quantitative phase imaging. Opt. Lasers Eng..

[21] Praveen P.A., Arockiaraj F.G., Gopinath S., Smith D., Kahro T., Valdma S.-M., Bleahu A., Ng S.H., Reddy A.N.K., Katkus T. (2022). Deep Deconvolution of Object Information Modulated by a Refractive Lens Using Lucy-Richardson-Rosen Algorithm. Photonics.

